# Noninvasive molecular diagnosis of craniopharyngioma with MRI-based radiomics approach

**DOI:** 10.1186/s12883-018-1216-z

**Published:** 2019-01-07

**Authors:** Xi Chen, Yusheng Tong, Zhifeng Shi, Hong Chen, Zhong Yang, Yuanyuan Wang, Liang Chen, Jinhua Yu

**Affiliations:** 1 Department of Electronic Engineering, Fudan University, Shanghai, China; 2 Department of Neurosurgery, Huashan Hospital, Fudan University, Shanghai, China; 3 Department of Pathology, Huashan Hospital, Fudan University, Shanghai, China; 4 Department of Radiology, Huashan Hospital, Fudan University, Shanghai, China

**Keywords:** Craniopharyngioma, Molecular diagnosis, Radiomics approach, Non-invasiveness, Machine learning

## Abstract

**Background:**

Frequent somatic mutations of BRAF and CTNNB1 were identified in both histological subtypes of craniopharyngioma (adamantinomatous and papillary) which shed light on target therapy to cure this oncogenic disease. The aim of this study was to investigate the noninvasive MRI-based radiomics diagnosis to detect BRAF and CTNNB1 mutations in craniopharyngioma patients.

**Methods:**

Forty-four patients pathologically diagnosed as adamantinomatous craniopharyngioma (ACP) or papillary craniopharyngioma (PCP) were retrospectively studied. High-throughput features were extracted from manually segmented tumors in MR images of each case. The modifications-robustness in region of interests and Random Forest-based feature selection methods were adopted to select the most significant features. Random forest classifier with 10-fold cross-validation was applied to build our radiomics model.

**Results:**

Four features were selected to make pathological diagnosis between ACP and PCP with area under the receiver operating characteristic curve (AUC) of 0.89, accurancy (ACC) of 0.86, sensitivity (SENS) of 0.89 and specificity (SPEC) of 0.85. The other two features were applied to estimate BRAF V600E mutation with AUC of 0.91, ACC of 0.93, SENS of 0.83 and SPEC of 0.97. Accurate predication of CTNNB1 mutation by three selected features was realized with AUC of 0.93, ACC of 0.86, SENS of 0.86 and SPEC of 0.86.

**Conclusions:**

We developed a reliable MRI-based radiomics approach to perform pathological and molecular diagnosis in craniopharyngioma patients with considerably accurate prediction, which could offer potential guidance for clinical decision-making.

**Electronic supplementary material:**

The online version of this article (10.1186/s12883-018-1216-z) contains supplementary material, which is available to authorized users.

## Background

Craniopharyngioma (CP) is a rare central nervous system tumor with incidence of 0.19/100, 000 every year in America and accounting for 0.8% of brain tumor [1], namely, fifty craniopharyngiomas patients would roughly correspond to six thousands brain tumors and a population of twenty-six millions. In WHO criterion, CP is defined as a benign tumor with two histological subtypes called ACP and PCP [2]. Due to its anatomic proximity to vital brain structures like brainstem, hypothalamus, pituitary stalk and optic nerves, devastating neurological dysfunction will be caused with high mortality and morbidity, which makes it so-called “behavior malignant tumor” [3, 4]. Surgical resection followed by radiation is the standard therapy. Even though, in some large-sized or complex tumors, radical eradication is hardly achieved and tends to cause severe post-operative complications and death as well. Craniopharyngioma was one of most challenging tumor for every neurosurgeon [5], and the quite low incidence data also presented difficulties in data collection for therapy study.

Recently, thanks to high throughput sequencing technology, 2 activating oncogenic driver BRAF and CTNNB1 were revealed to show highly frequent somatic mutations in CPs [6–8]. Further studies demonstrated strong correlation between the gene mutations and the pathological subtypes, BRAF V600E mutation in PCP and CTNNB1 mutations in ACP which were mutual exclusive [4, 9–11]. By targeting BRAF, BRAF/MEK inhibitor was proved to be effective in recurrent CPs in 2 clinical case reports worldwide [12, 13]. Referring to these findings, neo-adjuvant target therapy plus minimal-invasive microsurgery was regarded to be a new strategy to cure large-sized or complex CPs.

However, “Neo-adjuvant Therapy” means molecular diagnosis should be confirmed before operation. At present, the only way to realize pre-operative molecular diagnosis is doing genetic sequencing in peripheral blood test, and this method is technically inconvenience and expensive [14, 15]. Radiomics approach which refers to revealing the deep correlation between the medical images and the underlying information including gene, protein, physiology and pathology by turning the medical images into the minable high-throughput features, provides potential possibility to solve this problem. Our group has previously proposed radiomics models to predict genotypes of gliomas [16, 17]. In this study, we intended to propose an effective noninvasive radiomics models for the estimation of BRAF and CTNNB1 mutations in craniopharyngiomas.

## Methods

### Patient population

The study was approved by the local ethics committee of Huashan Hospital. We retrospectively reviewed medical records of patients who underwent surgery for craniopharyngioma in single neurosurgical institution (Huashan Hospital, Fudan University) from 2015 to 2017. Forty-four patients diagnosed of craniopharyngioma pathologically were enrolled with complete pre-operation MRI data. Central review and histological subtyping were performed by two individual neuropathologists. In our cohort, there were 29 male and 15 female patients, with 9 pediatric patients (mean age 12.1 years, range 5–17 years) and 35 adult patients (mean age 43.9 years, range 19–66 years). Among all patients, 32 cases were primary craniopharyngiomas, and 12 cases were recurrent tumors or with ventriculoperitoneal shunt history.

### Histological and molecular diagnosis

Adamantinomatous craniopharyngiomas or papillary craniopharyngiomas of 44 cases were diagnosed independently by two individual neuropathologists based on H&E review. Immunostaining for BRAF V600E (Spring Biosciences, USA, VE-1, 1:40) and β-Catenin (BD Biosciences, USA, clone 14, 1:800) was performed by Ventana NexES Staining System in a proportion of patients. BRAF V600E on exon 15 and CTNNB1 mutation on exon 3 were detected and analyzed by sanger sequencing as previously reported [18].

### Imaging data features acquisition and analysis

High resolution preoperative T1-MPRAGE MR images were acquired using a Magnetom Trio 3 T (Siemens) scanner. The size of MR images was 512 × 448, and MR images were stored as 16-bit unsigned integer. All images were acquired by using following parameters: pixel spacing = 0.488 mm, slice thickness = 1 mm, repetition time = 1900 ms, echo time = 2.93 ms, inversion time = 900 ms, flip angle = 9°. Two experienced radiologists blinded to patients’ clinical characteristics first segmented tumor lesions in MR images independently and then culminated in consensus on their discrepancies. High-throughput features extraction, feature selection and classification were sequentially performed to build the radiomics model. The details of each process are present below.

#### Location features

The location of craniopharyngioma was considered highly correlating with its genotype [18]. Instead of visually determining whether the tumor is completely suprasellar as previous study [18], a more quantitative location evaluation method was used in this study. To reduce the anatomical variability among individual brain, Statistical Parametric Mapping package (SPM) was utilized to register the MR images into the normalized Montreal Neurological Institute(MNI) space [19]. The MNI brain atlas is then divided into 116 anatomical volumes of interest (AVOIs) by Anatomical Automatic Labeling (AAL) [20]. After brain MR images of 44 patients are registered spatially and normalized into MNI space, the three-dimensional distance and Euclidean norm among centers of 116 AVOIs and center of tumor in MNI space are calculated to serve as the location features, which leads to 464(116*4) features.

#### Intensity, shape, texture and wavelet features

In addition to 464 location features, another 555 high-throughput features measuring intensity, shape, texture and wavelet are generated from the segmented volumes. The dimensions of features in each category are 21, 15, 39 and 480 respectively. Intensity features are applied to describe the intensities distribution of pixels within the tumor volumes. Shape features typify the morphological structure of the tumor. Texture features reflect the properties of tumor arrangement spatially. Wavelet features quantify the intensity and texture of each MR image in different eight frequency sub-bands and various feature orientations. All of 555 features have generally been used in previous radiomics studies [21–24]. The total of 1021 features including 464 location features, the above 555 high-throughput features and two clinical features (patients’ gender and age at diagnosis) are demonstrated in Additional file 1. These feature-extracting algorithms are calculated using the Matlab R2017a version (MathWorks).

### Feature selection

Among the 1021 features, a large quantity of them was highly redundant, which brought difficulties in the classification and increased the computational complexity [25]. To identify a small number of features that are optimal for classification of pathological subtypes and genetic mutational status, we proposed a three-stage feature selection method to gradually select the most relevant features.

In the first stage, we modified the manual segmented regions of tumor lesions by eight scenarios as follows: (a) horizontal translation by 2 pixels; (b) horizontal and vertical translation by 2 pixels; (c) 1° rotation; (d) 5° rotation; (e) combining modifications a, b and c; (f) combining modifications a, b and d; (g) enlarging by 1 pixel along radial lines; and (h) shrinking by 1 pixel along radial lines. Then we assessed modifications-robustness in region of interests of high-throughput texture features using the intraclass correlation coefficient (ICC) [26]. High-throughput features were selected as robust variables with ICC greater than 0.8.

In the second stage, we enrolled robust texture features, location features and clinical features, and adopted a Random Forest classification model using the 10-fold cross validation [27]. In every fold, all the features were ranked according to their contribution to decreasing the impurity, and the top 25% were preserved as remarkable features. We performed the model construction in the datasets for 100 bootstrap repetition, namely, we obtained 1000 lists of remaining features. Then, we sorted the features descendingly based on their respective sum of contribution in the 1000 ranking lists.

In the third stage, a sequential forward selection strategy was applied to carefully select a small group of significant features in compliance with the contribution ranking. We still adopted a Random Forest classification model to evaluate the predictive accuracy for candidate subset of features using the 10-fold cross validation. The sequential forward selection strategy is a feature selection strategy that sequentially adds one feature from top to bottom according to the contribution ranking. The classification accuracy was recorded for each subset of features and the selection process stopped when all features were added. The subset of features with the highest classification accuracy was finally selected.

### Classification

In this paper, the selected features were fed into an outer Random Forest to build the ultimate prediction model of pathological subtypes, BRAF and CTNNB1 mutational status. The performance of classification model was evaluated and validated by employing the 10-fold cross validation model. Several indexes, containing accuracy(ACC), sensitivity(SENS), specificity(SPEC), positive predictive value(PPV), negative predictive value(NPV), Matthew’s correlation coefficient (MCC), out-of-bag (OOB) score and the area under the receiver operating characteristic curve (AUC), were obtained as metrics to assess quantitative discrimination performance of the radiomics model in the classification of pathological subtypes and genetic mutational status.

In 10-fold cross-validation, the original dataset was randomly partitioned into ten equal sized subsets. Of the ten subsamples, nine subsamples were used as training data for model construction and the remaining one subsample was retained as the validation data for testing the model. The cross-validation process was then repeated ten times, with each of the ten subsets used exactly once as the validation data. Therefore, classification results of cases in every validation fold could be obtained for the all trees in the relevant random forest model. Then we calculated the proportion of positive prediction results size to the number of trees as scores for every validation sample and the positive result was defined by ACP, BRAF mutation or CTNNB1 mutation. Finally, we performed this technique in the all folds and acquired the scores of all cases. The ROC curve could be plot based on the score variables. The threshold of score variables was set to 0.5, which means that the category with the most votes of trees is specified as the final classification result in the random forest. The patients with score variable above 0.5 were classified as positive cases. Hence, the prediction results of all patients were acquired. For the pathological subtypes model, sensitivity is defined for ACP and specificity is defined for PCP. For the genetic mutational status models, sensitivity and specificity are defined to estimate the mutation and wild type, respectively. The measurement indexes could be calculated based on the ROC curve and prediction results ultimately. Using all cases rather than 10-fold cross validation to construct the prediction models, we can acquire the generalization accuracy (OOB score).

In addition, radiomics-based prognostic nomograms were developed based on selected features, and the discriminative ability of prognostic models could be measured using Harrell’s C-index. The value of the C-index ranges from 0.5, which indicates no discriminative ability, to 1.0, which indicates perfect ability to distinguish pathological subtypes and genetic mutational status.

### Statistical analysis

Mann–Whitney U test and Fisher exact test were used to evaluate whether age, gender and pathological types had statistical differences between different gene statuses. The rms package was used for nomograms, and the Hmisc package was used for calculation of C-index in R software. Statistical significance was set as *p* < 0.05. All statistical analyses were performed by using SPSS software version 22.0 (IBM Corp.) and R software.

## Results

### Clinical, genetic, and pathological findings

Including forty-four patients in our study, we stratified all the cases into three categories based on mutational status included BRAF mutation group, CTNNB1 mutation group and not detected (BRAF and CTNNB1 gene status are both wild types.) group. Distribution of gender, age, pathological subtypes and mutational profile was summarized in Table 1. There was a significant relationship between age and mutational profile (*p* = 0.002). Six (60.0%) of ten cases with not detected status was observed in pediatric patients while nineteen (86.4%) of twenty-two CTNNB1 mutant cases struck adult patients and all twelve (100%) BRAF mutant cases were adults. This result was also in accordance with the previous study [4]. In addition, pathological types also had a strong correlation with mutational profile (*p* < 0.001), and all twelve (100%) tumors were classified as papillary craniopharyngiomas in the BRAF mutation group while eighteen (81.8%) of twenty-two tumors were classified as adamantinomatous craniopharyngiomas in the CTNNB1 mutation group. For not detected cases, eight (80.0%) of ten tumors belonged to adamantinomatous craniopharyngiomas. BRAF V600E mutation and CTNNB1 mutation were mutual exclusive in all cases. Mutational profile was not changed between primary and paired recurrent craniopharyngiomas (*p* = 1.000) in Table 1. Distinct immunostaining patterns of BRAF V600E and β-Catenin in different histopathological subtypes were seen in Additional file 2. Illustration of BRAF and CTNNB1 mutation profile can be seen in the Additional file 3.

**Table 1:** Demographics characteristics stratified by the BRAF and CTNNB1 mutations status in the primary patients and recurrent patients

	BRAF Mutant *N* = 12		CTNNB1 Mutant *N* = 22		Not Detected *N* = 10		*P* Value
Dataset	PP(*N* = 9)	RP(*N* = 3)	PP(*N* = 15)	RP(*N* = 7)	PP(*N* = 8)	RP(*N* = 2)	1.000
Gender							0.770
Male (%)	9 (75.0)		14 (63.6)		6 (60.0)		
	7	2	12	2	5	1	
Female (%)	3 (25.0)		8 (36.4)		4 (40.0)		
	2	1	3	5	3	1	
Age(year)							0.002
Mean ± SD	47.0 ± 9.9		37.9 ± 15.9		24.9 ± 18.8		
	49.7 ± 8.65	39.0 ± 10.8	39.8 ± 15.9	33.9 ± 16.3	28.0 ± 19.9	12.5 ± 6.4	
>18 (%)	12 (100.0)		19 (86.4)		4 (40.0)		
	9	3	13	6	4	0	
<18 (%)	0 (0.0)		3 (13.6)		6 (60.0)		
	0	0	2	1	4	2	
Pathology							<0.001
ACP (%)	0 (0.0)		18 (81.8)		8 (80.0)		
	0	0	13	5	6	2	
PCP (%)	12 (100.0)		4 (18.2)		2 (20.0)		
	9	3	2	2	2	0	

Abbreviations: *PP* Primary patients, *RP* Recurrent patients, *SD* Standard deviation, *ACP* Adamantinomatous type, *PCP* Papillary type

### Tumor segmentation

**Fig. 1:**
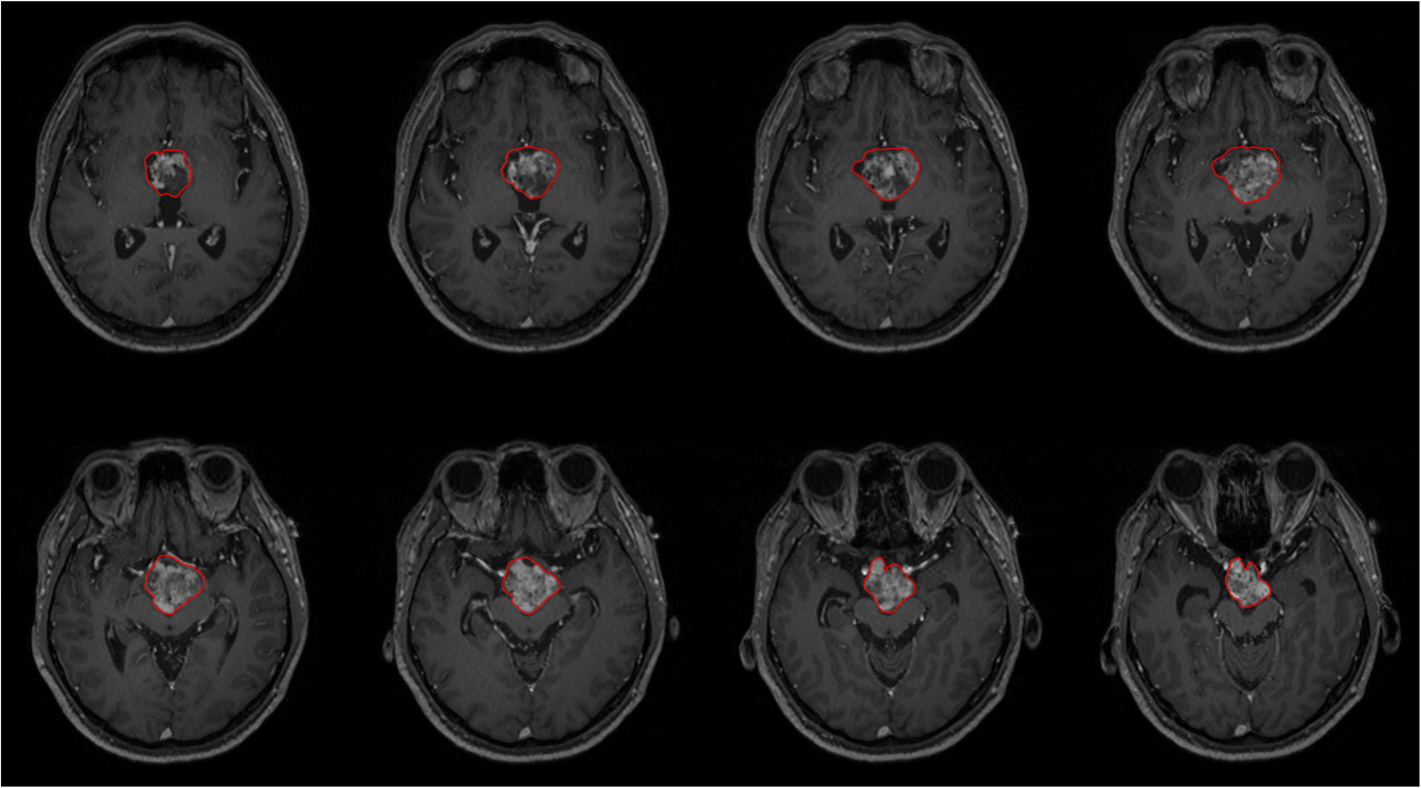
Tumor segmentation results of eight representative axial T1-MPRAGE MR images obtained obtained from one BRAF mutant case. In each image, the area surrounded by red line indicated the tumor

The tumor segmentation results were shown in Fig. 1, including eight representative axial T1-MPRAGE MR images obtained from one BRAF mutant case. In each image, the area surrounded by red line indicated the tumor.

### Data partitioning

Thirty-two primary craniopharyngiomas patients were used as the main cross validation dataset. We selected the most significant features of the main dataset for prediction models. The main radiomics models were constructed based on the main dataset. Another 12 recurrent or treated cases were added into the main database as extensional database.

As the main radiomics models were used for pathological subtypes and gene status prediction of primary craniopharyngiomas, using the 12 recurrent or treated patients as a test database could not accurately assess model performance. Besides, the number of additional patients is not enough for recurrent model construction. The additional cases are also imbalanced such as only three BRAF mutation patients (Table 1). Because the prediction on primary and recurrent mixed cases was more in line with clinical status, the previous selected features in the primary database were directly used for construction of ultimate classification model in extensional database to test their extensiveness on recurrent or treated cases skipping the feature selection procedure. More reliable prediction results could be obtained combining the additional cases with main dataset.

### Radiomics model construction based on main dataset

**Fig. 2:**
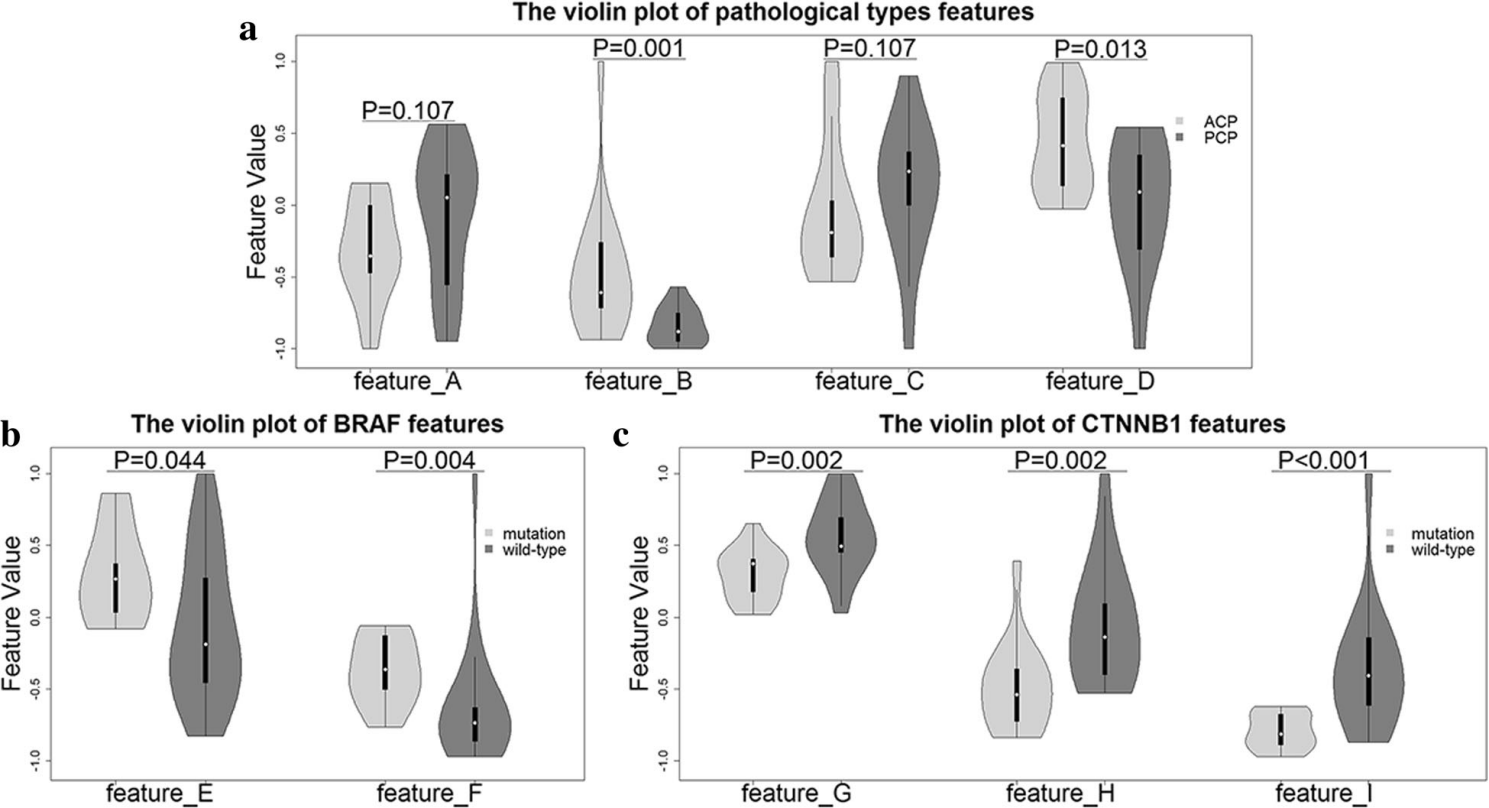
The violin plot of discriminative features. **a** Dissimilarity of LLL decomposition (feature A), kurtosis (feature B), root mean square (feature C) and compactness (feature D); **b** small zone emphasis of HHL decomposition (feature E) and short run low gray-level emphasis (feature F); **c** h-skewness of HLL decomposition (feature G), h-mean of HHH decomposition (feature H) and short run low gray-level emphasis (feature I). Mann-Whitney’ test was used to assess significance of difference and *p* value was put above the violin plot of each feature

Three hundred and seventy one texture features had an ICC greater than 0.8 on the basis of digital algorithmic modification in Additional file 4. After the first stage of feature selection method, we obtained eight hundred and thirty seven preliminary features in total. Then using the random-forest based feature selection method, several significant features were selected and involved in the further prediction step of pathological subtypes and genetic mutational status. We obtained four features for pathological subtypes discrimination, two features for BRAF V600E mutational prediction and three features for CTNNB1 mutational status estimation, respectively. Three violin plots, which combine the box plots and kernel density plots, are drawn to demonstrate difference of selected radiomics features within three taxonomies including pathological subtypes, BRAF and CTNNB1 genetic mutational status (Fig. 2). In pathological subtypes discrimination, four selected features are called dissimilarity of LLL decomposition (feature A), kurtosis (feature B), root mean square (feature C) and compactness (feature D).In BRAF V600E mutational prediction, two selected features are known as small zone emphasis of HHL decomposition (feature E) and short run low gray-level emphasis (feature F). In CTNNB1 mutational status estimation, three selected features are named h-skewness of HLL decomposition (feature G), h-mean of HHH decomposition (feature H) and short run low gray-level emphasis (feature I). All features were normalized to the − 1 to 1 range. The calculations of these features are presented in Additional file 1.

**Fig. 3:**
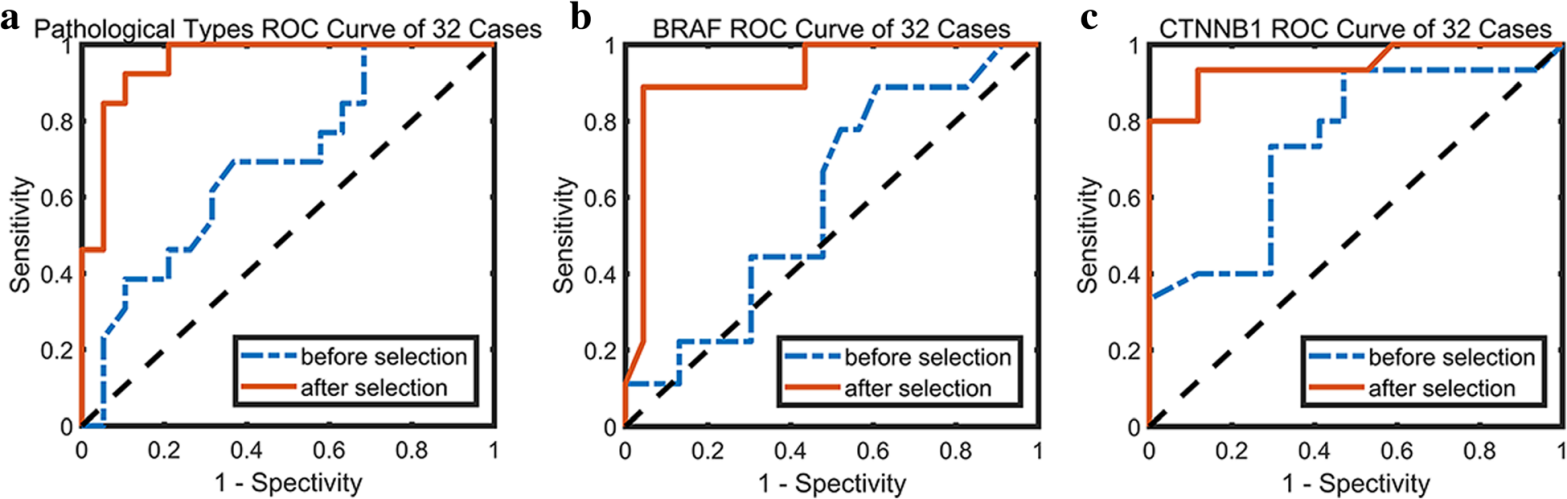
ROC curves of prediction before and after feature selection based on main dataset. **a** Pathological subtypes ROC curve; **b** BRAF gene ROC curve; **c** CTNNB1 gene ROC curve

As shown in Table 2, our proposed radiomics method achieved respectable performance in pathological subtypes and genetic mutational status classification on 10-fold validation cohort. Craniopharyngioma pathological subtypes were discriminated with AUC of 0.96, ACC of 0.91, SENS of 0.92 and SPEC of 0.89. BRAF V600E mutational status was estimated with AUC of 0.92, ACC of 0.94, SENS of 0.89 and SPEC of 0.96. CTNNB1 mutational status was predicted with AUC of 0.95, ACC of 0.91, SENS of 0.93 and SPEC of 0.88. Figure 3 shows the receiver operating characteristic curve (ROC) of radiomics classification model before and after selection.

**Table 2:** Pathological types, BRAF gene and CTNNB1 gene status differentiation performance in different datasets

	Dataset		AUC	ACC	SENS	SPEC	PPV	NPV	MCC	OOB
Pathological types	*n* = 32	BS	0.69	0.63	0.38	0.79	0.56	0.65	0.19	0.66
		AS	0.96	0.91	0.92	0.89	0.86	0.94	0.81	0.91
	*n* = 44	–	0.89	0.86	0.89	0.85	0.80	0.92	0.73	0.85
BRAF gene	*n* = 32	BS	0.59	0.69	0.11	0.91	0.33	0.72	0.04	0.63
		AS	0.92	0.94	0.89	0.96	0.89	0.96	0.85	0.91
	*n* = 44	–	0.91	0.93	0.83	0.97	0.91	0.94	0.83	0.93
CTNNB1 gene	*n* = 32	BS	0.74	0.72	0.73	0.71	0.69	0.75	0.44	0.66
		AS	0.95	0.91	0.93	0.88	0.88	0.94	0.81	0.88
	*n* = 44	–	0.93	0.86	0.86	0.86	0.86	0.86	0.73	0.86

Abbreviations: *BS* Before selection, *AS* After selection

In the feature selection procedure, all variables including the location features were enrolled to select the optimal features for prediction. There were no location features included in the optimal features subset. However, due to the correlations between features [25], the most significant features subset will change if some features are removed from the feature set including all variables even though the removed features are not in the set of selected features. Although our ultimate radiomics models did not contain any location feature, these location features contributed to selecting the most significant features subset. In our experiments, the performance of the model decreased a bit without considering location features.

**Fig. 4:**
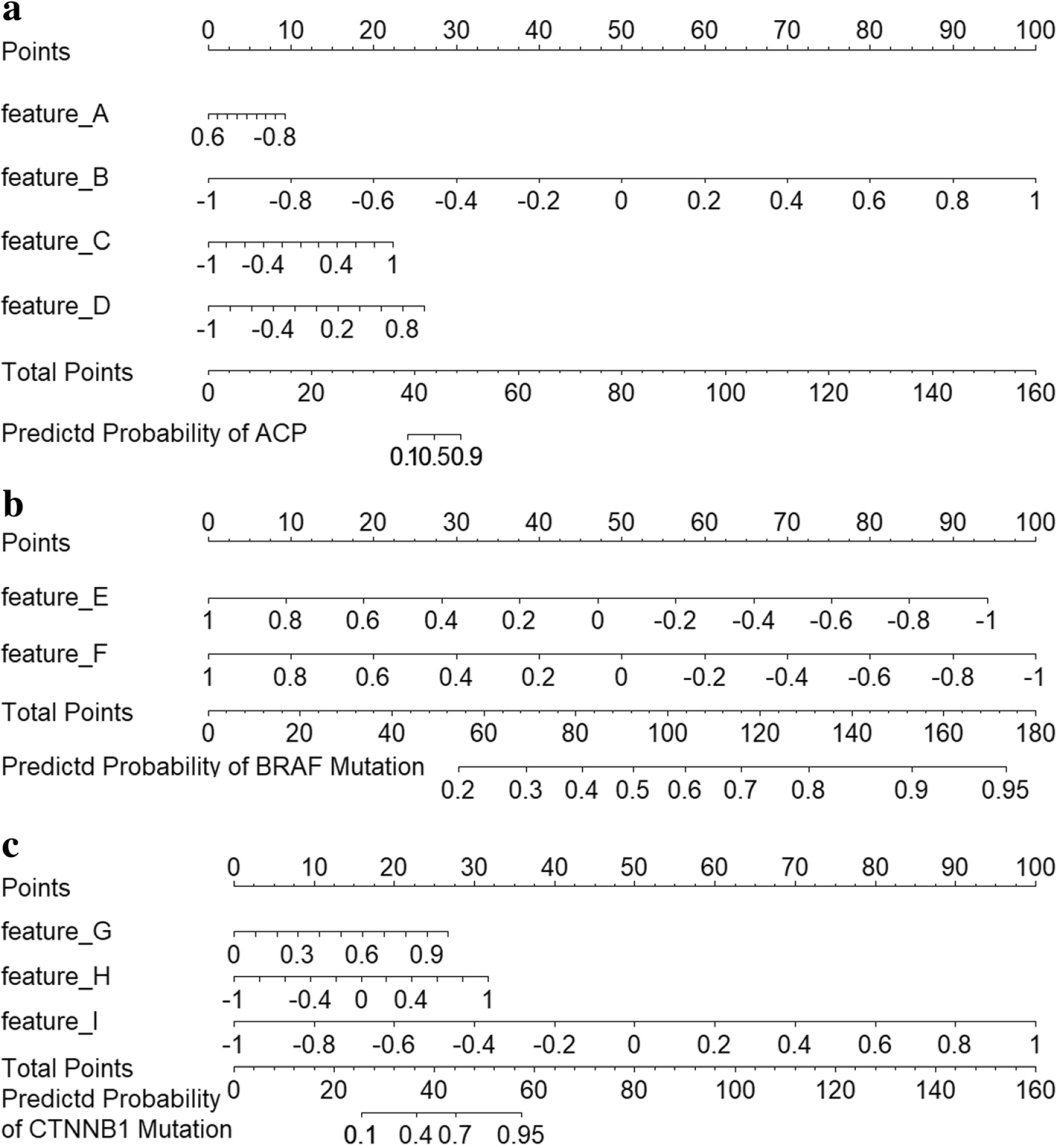
Three radiomics nomograms integrate four discriminative features in main dataset. **a** Feature A, feature B, feature C and feature D of pathological subtypes classification; **b** feature E and feature F of BRAF gene prediction; **c** feature G, feature H, and feature I of CTNNB1 gene estimation

To evaluate the weights of involved features in classification model, three nomograms were developed as individualized tools in Fig. 4. The selected radiomics features shows good ability to distinguish pathological subtypes (C-index of 0.819) and genetic mutational status (C-index of 0.810 for BRAF gene and C-index of 0.912 for CTNNB1 gene). To use the nomogram, find the predictor points on the uppermost point scale that correspond to each patient radiomics feature and add them up. The total points projected to the bottom scale indicate the probability of ACP, BRAF mutation or CTNNB1 mutation. Namely, the points of selected features could represent their weights in corresponding radiomics model.

Although we extracted a very large and complex set of features to describe the whole tumor regions, only a few features were put into the final classification model after the feature selection. That is to say, very few feature parameters were eventually used for the model construction. Moreover, the Random Forest-based classification method could reduce the risk of overfitting as well. Random forests do not overfit as more trees are added, and produce a limiting value of the generalization error [27]. Via limiting the number of splits or the size of nodes for which splitting is allowed, gains could be realized to avoid overfitting [28].

### Radiomics model construction based on extensional dataset

**Fig. 5:**
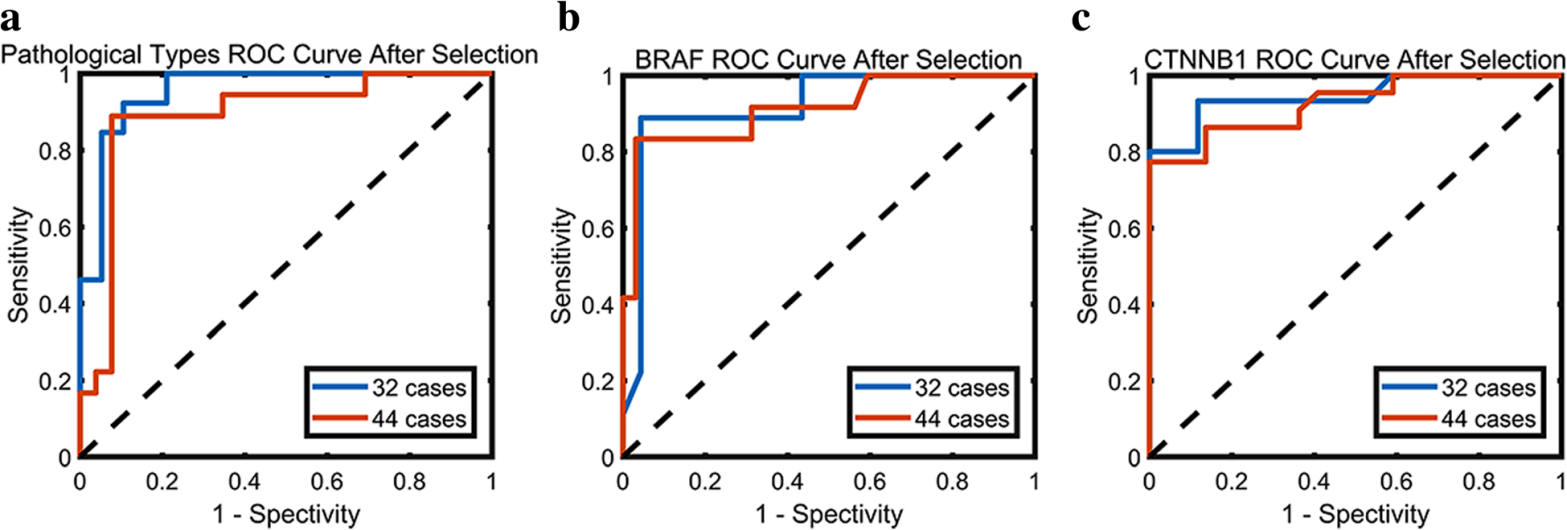
ROC curves of estimation after feature selection based on main dataset and extensional dataset. **a** Pathological types ROC curve; **b** BRAF gene ROC curve; **c** CTNNB1 gene ROC curve

To verify the stability of our proposed radiomics method, 12 cases who had recurrent tumors or with previous history of ventriculoperitoneal shunt were added into the main cross validation dataset to further test the performance of the method. The discriminative features, which were selected from the previous radiomics model based on main dataset, were included in the following construction of Random Forest classification model using the extensional dataset on 10-fold validation cohort. Craniopharyngioma pathological subtypes were discriminated with AUC of 0.89, ACC of 0.86, SENS of 0.89 and SPEC of 0.85. BRAF V600E mutational status was estimated with AUC of 0.91, ACC of 0.93, SENS of 0.83 and SPEC of 0.97. CTNNB1 mutational status was predicted with AUC of 0.93, ACC of 0.86, SENS of 0.86 and SPEC of 0.86. Table 2 lists the classification performance difference in different datasets using the same features, which reveals a bit drop in performance within treated or recurrent cases. Figure 5 exhibits the ROC of the classification model in main dataset and further validation dataset after feature selection, and the ROC of different datasets are very close to each other.

## Discussion

Craniopharyngiomas are locally aggressive parasellar tumors with relatively low incidence compared to other brain tumors [1]. In our cohort, forty –four patients were enrolled, which corresponded to six thousands brain tumors. Some of patients were present with profound neurological deficits; meanwhile, total resection was not performed in every cases. Recent genomic findings provided potential targets for anti-cancer treatment in craniopharyngiomas. Researchers identified frequent somatic mutation of BRAF V600E and CTNNB1 in CPs [6, 7]. These two mutations were mutual exclusive in different subtypes defined pathologically as adamantinomatous and papillary [4, 9–11]. Researchers reported that such kind of genetic alteration were observed in 76–96% CPs [6, 7, 29]. In our small sample size cohort, the mutational ratio of both BRAF and CTNNB1 were a little bit lower compared to previous reports. This result may lead to the reason that sanger sequencing used in our study was less sensitive to next generation sequencing(NGS) in detecting BRAF and CTNNB1 mutation. However, Sanger sequencing is more simple and cost-effective in basic molecular lab. It was still the first choice in regular clinical practice.

According to successful experience in BRAF inhibitors targeting BRAF mutant cancers like melanoma [30], two patients of recurrent CPs with BRAF V600E mutation were subscribed with BRAF/MEK inhibitors [12, 13]. Target therapy worked well in a short period resulting in significant tumor size decrease. These clinical outcomes were inspiring and came to the idea of neo-adjuvant target therapy for large-sized or complex tumors. Neurosurgeons can perform safe surgery for CPs with more possibility of radical resection if this target therapy really works. Before operation, accurate molecular diagnosis is crucial to the administration of target therapy. In Brastianos et al. report, they announced BRAF mutation could be detected in patients’ peripheral blood samples [13]. However, we could not duplicate their result in our lab, and since now, not so many data support liquid biopsies for primary benign brain tumor patients. We speculated that existence of brain-blood-barrier is the main reason for the failure of liquid biopsy. Even if it works, expensive price and sophisticated lab setting-up will be major problems. That is why we want to develop a MRI-based radiomics approach for noninvasive prediction of genetic alterations.

In our study, a radiomics-based approach was proposed to classify pathological subtypes and detect genes mutational status. Several successful precedents for reference have been shown in recent radiomics studies about gene detection of brain tumors. A primary cohort consisting of 110 patients pathologically diagnosed with grade II glioma was retrospectively studied [17]. In LOOCV, the noninvasive isocitrate dehydrogenase 1 (IDH1) status estimation presented an estimation AUC of 0.86, ACC of 0.80, SEN of 0.83 and SPEC of 0.74. Deep learning-based radiomics (DLR) was developed on a dataset of 151 patients with low-grade glioma with multiple modalities of magnetic resonance (MR) images for predicting the mutation status of IDH1, and achieved ACC of 0.91 and AUC of 0.96 [16]. Yue et al. proposed a novel diagnostic criterion of BRAF mutation and wild type in craniopharyngiomas with SENS of 1.00 and SPEC of 0.91 [18]. In our study, high-throughput features applied into our radiomics model are extracted quantitatively and noninvasively on medical images following the well-established standard to describe the structural heterogeneity of tumors. In contrast, diagnostic criterion proposed by Yue et al. is confirmed empirically, which is notwithstanding worth considering such as tumor location. In this paper, we measured the tumor location with Anatomical Automatic Labeling (AAL) by spatial registration and normalization of brain images into MNI space. Since AAL is designed for cerebral hemispheres and the sellar regions - routine lesions of craniopharyngiomas [20] - are anatomical areas rather than cerebral hemispheres, the location features were quantified using three-dimensional distance vector and Euclidean norm among center of each anatomical volume of interest (AVOI) and tumor.

Although the high-throughput features analysis exploits the ability of characterizing the whole tumor regions, it is evident that radiomics features extraction provides a very large and complex set of data, which presents high correlation among them and results in risk of overfitting. Thus, it is necessary to reduce the number of features to develop an estimation model. Based the Random Forest-based feature selection and classification method, several significant features are obtained for model construction as this approach could handle the problem of overfitting [27, 28].

In the main dataset, our proposed radiomics method selects four radiomics features and classifies PCPs and ACPs with an AUC curve of 0.96 and ACC of 0.91. BRAF V600E mutation and wild-type in craniopharyngiomas are discriminated with an AUC curve of 0.92 and ACC of 0.94 using two radiomics features while CTNNB1 mutation and wild-type in craniopharyngiomas are distinguished with an AUC curve of 0.95 and ACC of 0.91 based on three radiomics features. Although the use of selected features could contribute to the performance of radiomics model, they are challenging to decipher with the naked eye. Moreover, it remains a significant obstacle to ensure that clinicians expert in gleaning detailed information from imaging features. However, these undiscerned features contain more detailed information of morphological structure and texture about the tumor. Therefore, by incorporating these radiomics features, a radiomics-based model can assist doctors in accurately identifying patients with pathological subtypes and genetic mutational status. Besides, textural features that are more effective for pathological subtypes and genetic mutational status estimation could be selected by employing MR intensity standardization [31]. Three nomograms are developed as individualized tools to assess the weights of integrated features in classification, and the selected radiomics features reveals adequate discrimination in pathological subtypes (C-index of 0.819) and genetic mutational status (C-index of 0.810 for BRAF gene and C-index of 0.912 for CTNNB1 gene). Adding 12 treated patients to main dataset, the performance of radiomics model has a bit drop in pathological subtypes classification and has little drop in BRAF V600E and CTNNB1 mutational prediciton, which demonstrates the viability and effectiveness of our radiomics method to construct prediction model in pathological types and gene status in despite of tumor changes after treatment. Compared with Sanger sequencing and NGS, the radiomics method is noninvasive, higher sensitivity and lower detection cost that MR images of patients are only required.

### Limitations

Our study has some limitations. Firstly, the study retrospectively was based on patients with definite pathological diagnosis. However, other tumors that occur in the sellar regions as craniopharyngiomas, including pituitary adenoma, Rathke’s cyst and germ cell tumor, may confuse surgeons preoperatively, and these tumors would be included in future studies. Secondly, the size of enrolled patients was not big enough. Since craniopharyngiomas is a rare disease, study based on multiple centers could enlarge the study dataset and improve the performance of radiomics model.

## Conclusion

In the current study, we developed and validated a novel MRI-based radiomics model for noninvasive prediction of pathological subtypes and genetic mutational status in patients with craniopharyngiomas. The radiomics nomograms fancilitated noninvasive estimation of pathological subtypes and genetic mutational status. The proposed radiomics model successfully stratified patients using the radiomics features extracted from MR images and selected using Random Forests algorithm, and could offer potential guidance for clinical decision-making.

## Additional files

Additional file 1: **Table S1.** Summary of 1021 radiomics features; the calculations of selected features in pathological subtypes and genetic mutational status prediction model. (PDF 259 kb)

Additional file 2: **Figure S1.** The histopathological findings in different groups. (PDF 540 kb)

Additional file 3: **Figure S2.** The examples of DNA electrophorograms for BRAF and CTNNB1 mutation. (PDF 257 kb)

Additional file 4: **Table S2.** High-throughput texture features which have an ICC greater than 0.8. (PDF 334 kb)

## Abbreviations

AAL: Anatomical Automatic Labeling
ACC: Accuracy
ACP: Adamantinomatous craniopharyngiomas
AUC: Area under the receiver operating characteristic curve
AVOI: Anatomical volume of interest
CP: Craniopharyngioma
CTNNB1: Catenin, beta-1
DLR: Deep learning-based radiomics
ICC: Intraclass correlation coefficient
IDH1: Isocitrate dehydrogenase 1
MCC: Matthew’s correlation coefficient
MNI: Montreal Neurological Institute
MR: Magnetic resonance
NPV: Negative predictive value
OOB: Out of bag
PCP: Papillary craniopharyngioma
PPV: Positive predictive value
RF: Random forest
SENS: Sensitivity
SPEC: Specificity
