# Supplementary material for: Noninvasive molecular diagnosis of craniopharyngioma with MRI-based radiomics approach
Source: BMC Neurol. 2019 Jan 7;19:6. doi: 10.1186/s12883-018-1216-z (PMC6322318; doi:10.1186/s12883-018-1216-z)
Supplement: Supplementary file 1 — Table S1. Summary of 1021 radiomics features; the calculations of selected features in pathological subtypes and genetic mutational status prediction model. (PDF 259 kb) [file 12883_2018_1216_MOESM1_ESM.pdf]

Additional file 1

Table S1. Summary of 1021 radiomics features

Feature category				Feature number
Location				464
Three-dimensional distance vector and Euclidean norm among center of 116 AVOIs and tumor in MNI space				
Intensity				21
energy	h-entropy	kurtosis	max	
mean absolute deviation	mean	media	min	
range	root mean square	skewness	standard deviation	
h-uniformity	variance	gauss-fitting-a	gauss-fitting-b	
gauss-fitting-c	h-mean	h-variance	h-skewness	
h-kurtosis				
Shape				15
compactness	compactness square	max-length	spherical disproportion	
sphericity	superficial area	surface to volume ratio	volume	
region to bounding-box ratio	max major-length	min minor-length	eccentricity	
orientation	solidity	Fourier-descriptors		
Texture				39
GLCM(gray-level co-occurrence matrix)				8
energy	contrast	entropy	homogeneity	
correlation	sum average	variance	dissimilarity	
GLRLM(gray-level run-length matrix)				13
short run emphasis	long run emphasis	GL nonuniformity	run-length nonuniformity	
run percentage	low GL run emphasis	high GL run emphasis	short run low GL emphasis	
short run high GL emphasis	long run low GL emphasis	long run high GL emphasis	GL variance	
run-length variance				
GLSZM(gray-level size zone matrix)				13
small zone emphasis	large zone emphasis	GL nonuniformity	zone-size nonuniformity	
zone percentage	low GL zone emphasis	high GL zone emphasis	small zone low GL emphasis	
small zone high GL emphasis	large zone low GL emphasis	large zone high GL emphasis	GL variance	
zone-size variance				
NGTDM(neighborhood gray-tone difference matrix)				5
coarseness	contrast	busyness	complexity	
strength				
Wavelet				480
LLL HLL LHL HHL LLH HLH LHH HHH decomposition				
Clinic				2
age	gender			
			Total feature number	1021

In this table, GL is gray-level.

We gained eight high frequency and low frequency sub-bands based on three orientations (X Y and Z coordinates) using the orthogonal Coiflets wavelet, which were called as LLL, HLL, LHL, HHL, LLH,

HLH, LHH and HHH. The selected features were calculated as following equations.

1) dissimilarity of LLL decomposition (feature A)

LLL decomposition represents the low frequency sub-band in all orientations. Dissimilarity based Gray-Level Co-Occurrence Matrix (GLCM) is defined in equation (1).

$$dissimilarity = \sum_{i=1}^{N_g} \sum_{j=1}^{N_g} |i-j| M(i,j) \quad (1)$$

N_g is the number of discrete intensity levels in the images. GLCM is defined as a matrix M(i,j;δ,θ) to indicate the relative frequency with intensity values of pixels (i and j) at the distance of δ in direction θ.

2) kurtosis (feature B)

Kurtosis is defined in equation (2). X represents one-dimensional vector which contains N elements and is transformed from the 3-D image volume, and $\bar{X}$ is the mean gray level of X.

$$kurtosis = \frac{\frac{1}{N}\sum_{i=1}^{N}(X(i)-\bar{X})^4}{\frac{1}{N}\sum_{i=1}^{N}(X(i)-\bar{X})^2} \quad (2)$$

3) root mean square (feature C)

Root mean square (RMS) is defined in equation (3).

$$RMS = \sqrt{\frac{\sum_{i=1}^{N} X(i)^2}{n}} \quad (3)$$

4) compactness (feature D)

Compactness is defined in equation (4). V and A represent volume and surface area respectively.

$$compactness = \frac{V}{\sqrt{\pi} A^{\frac{2}{3}}} \quad (4)$$

5) small zone emphasis of HHL decomposition (feature E)

HHL decomposition represents the sub-band of high frequency in XY orientations and low frequency in Z orientations. Small zone emphasis (SZE) based Gray Level Size Zone Matrix (GLSZM) is defined in equation (5).

$$SZE = \frac{\sum_{i=1}^{N_g} \sum_{j=1}^{N_r} \left[\frac{P(i,j)}{j^2}\right]}{\sum_{i=1}^{N_g} \sum_{j=1}^{N_r} P(i,j)} \quad (5)$$

GLSZM is defined as a matrix P(i, j) to indicate the areas of size j and gray level i. N_g is the number of discrete intensity levels in the images, and N_r is the number of different areas sizes.

6) short run low gray-level emphasis (feature F and I)

Short run low gray-level emphasis (SRLGE) based Gray Level Run Length Matrix based features (GLRLM) is defined in equation (6).

$$SRLGE = \frac{\sum_{i=1}^{N_g} \sum_{j=1}^{N_l} \left[\frac{Q(i,j;\theta)}{i^2 j^2}\right]}{\sum_{i=1}^{N_g} \sum_{j=1}^{N_l} Q(i,j;\theta)} \quad (6)$$

GLSZM is defined as a matrix Q(i,j;θ) to indicate the number of times j and gray level i appear consecutively in the direction θ. N_g is the number of discrete intensity levels in the images, and N_l is the number of different run lengths.

7) h-skewness of HLL decomposition (feature G)

HLL decomposition represents the sub-band of high frequency in X orientations and low frequency in YZ orientations. H-skewness is defined in equation (7). Let H represent the 3-D image histogram distribution. H contains N elements and $\bar{H}$ is the mean gray level of H.

$$h\_skewness = \frac{\frac{1}{N}\sum_{i=1}^{N}(H(i)-\bar{H})^3}{\left(\sqrt{\frac{1}{N}\sum_{i=1}^{N}(H(i)-\bar{H})^2}\right)^3} \quad (7)$$

8) h-mean of HHH decomposition (feature H)

HHH decomposition represents the high frequency sub-band in all orientations. H-mean is defined in equation (8).

$$h - mean = \bar{H} \quad (8)$$
