# Supplementary material for: Noninvasive molecular diagnosis of craniopharyngioma with MRI-based radiomics approach
Source: BMC Neurol. 2019 Jan 7;19:6. doi: 10.1186/s12883-018-1216-z (PMC6322318; doi:10.1186/s12883-018-1216-z)

Additional file 2

Fig S1. The histopathological findings in different groups. Histopathological examples of PCP (A,100X) and ACP (B,100X), the cytoplasmic staining pattern of BRAF V600E in PCP (C,400X), the immunonegative expression of BRAF V600E in ACP (D,400X), the nuclear and cytoplasmic staining pattern of β-Catenin in ACP (E,400X) and the membranous staining pattern of β-Catenin in PCPs (F,400X).

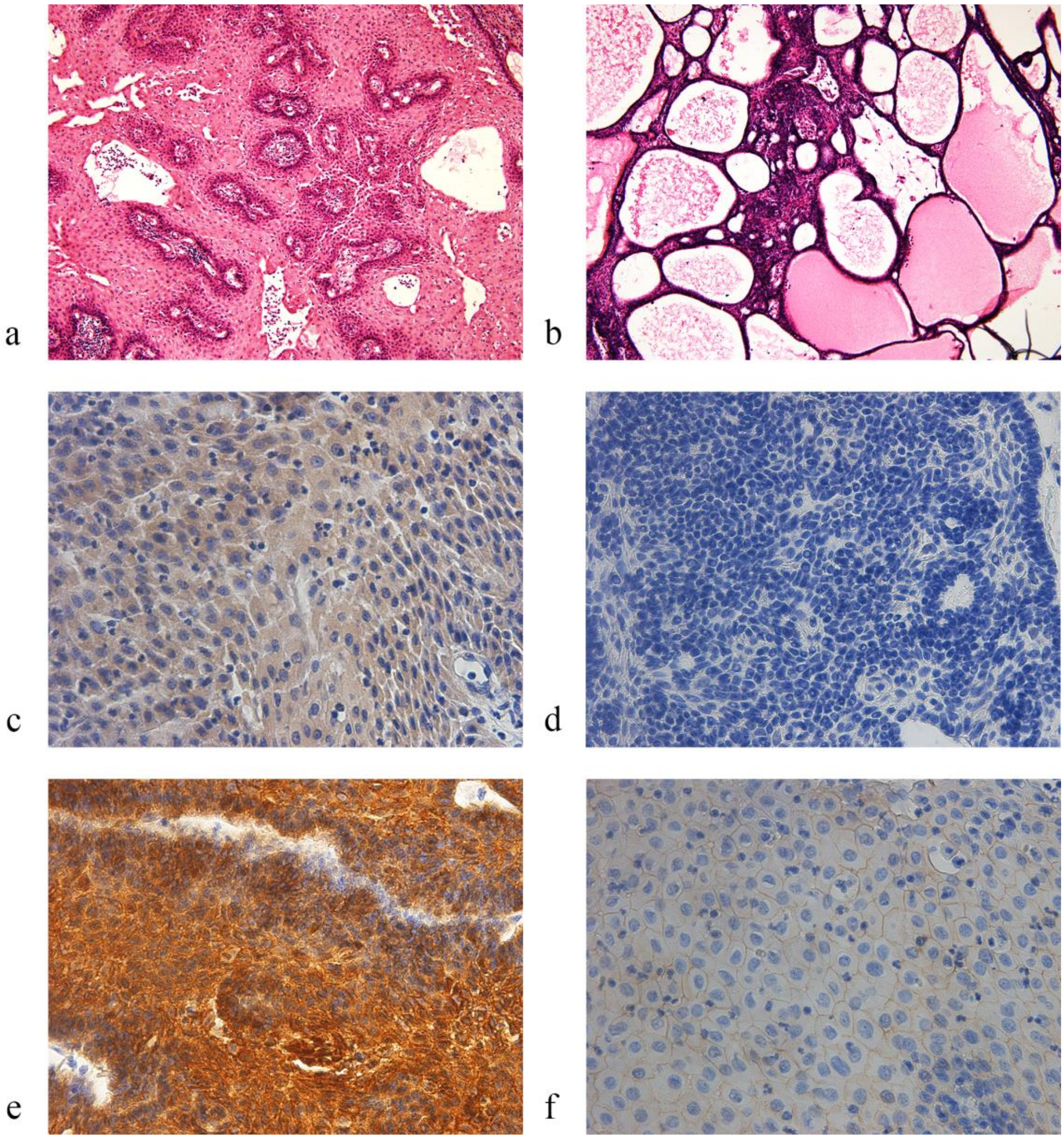

Supplement: Supplementary file 2 — Figure S1. The histopathological findings in different groups. (PDF 540 kb) [file 12883_2018_1216_MOESM2_ESM.pdf]
