# Supplementary material for: Noninvasive molecular diagnosis of craniopharyngioma with MRI-based radiomics approach
Source: BMC Neurol. 2019 Jan 7;19:6. doi: 10.1186/s12883-018-1216-z (PMC6322318; doi:10.1186/s12883-018-1216-z)

Additional file 3

Fig S2. The examples of DNA electrophorograms for BRAF and CTNNB1 mutation display as follows: BRAF V600E mutation (A), CTNNB1 D32H mutation (B), CTNNB1 D32N mutation (C), CTNNB1 S33C mutation (D), CTNNB1 S33F mutation (E), CTNNB1 G34R mutation (F), CTNNB1 S37C mutation (G), CTNNB1 S37F mutation (H), CTNNB1 T41A mutation (I), CTNNB1 S45F mutation (J) and CTNNB1 S45P mutation (K).

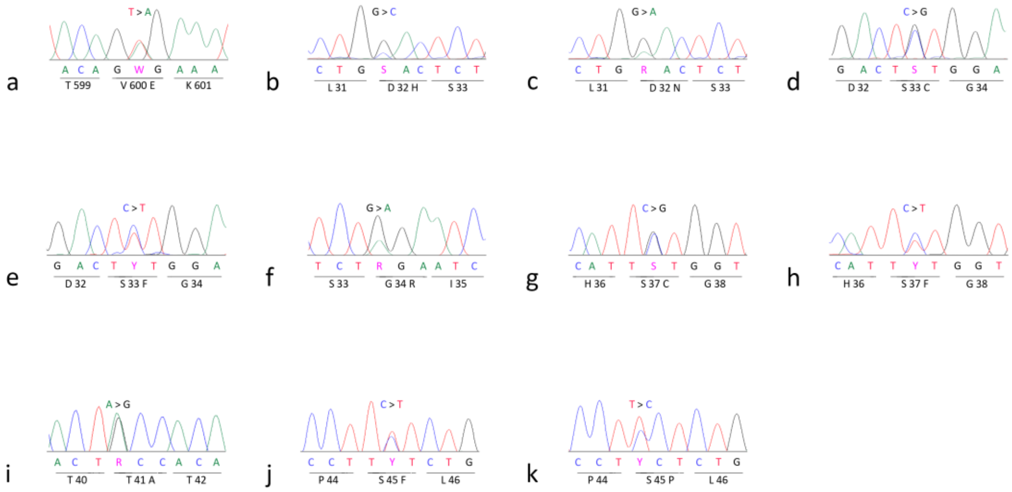

Supplement: Supplementary file 3 — Figure S2. The examples of DNA electrophorograms for BRAF and CTNNB1 mutation. (PDF 257 kb) [file 12883_2018_1216_MOESM3_ESM.pdf]
