# Supplementary material for: Noninvasive molecular diagnosis of craniopharyngioma with MRI-based radiomics approach
Source: BMC Neurol. 2019 Jan 7;19:6. doi: 10.1186/s12883-018-1216-z (PMC6322318; doi:10.1186/s12883-018-1216-z)
Supplement: Supplementary file 4 — Table S2. High-throughput texture features which have an ICC greater than 0.8. (PDF 334 kb) [file 12883_2018_1216_MOESM4_ESM.pdf]

Additional file 4

Table S2. High-throughput texture features which have an ICC greater than 0.8

High-throughput Texture Feature	Digital Algorithmic Modification ICC
energy	0.998044
h-entropy	0.955206
kurtosis	0.935106
max	0.847957
MAD	0.995835
mean	0.981303
media	0.99702
range	0.830866
skewness	0.997514
RMS	0.984957
SD	0.978279
h-uniformity	0.969404
var	0.975593
gauss-fitting-a	0.997573
gauss-fitting-b	0.99701
gauss-ficting-c	0.98093
h-mean	0.995837
h-var	0.975595
h-skewness	0.985012
h-kurtosis	0.935433
compactness	0.985527
compactness-square	0.983808
max-length	0.999355
Spherical disproportion	0.988589
sphericity	0.986095
superficial-area	0.997005
surface to volume ratio	0.998404
volume	0.997948
rbr	0.985459
max major-length	0.998871
min minor-length	0.995508
eccentricity	0.994232
orientation	0.922685
solidity	0.953145
Fourier-descriptors	0.987768
Energy	0.974205
Contrast	0.88653
Entropy	0.980849
Homogeneity	0.976006
Correlation	0.897248

SumAverage	0.948917
Variance	0.957513
Dissimilarity	0.940934
SRE	0.979477
LRE	0.965601
GLN	0.998797
RLN	0.996876
RP	0.9834
LGRE	0.865125
HGRE	0.88528
SRLGE	0.865512
SRHGE	0.874764
LRLGE	0.972989
LRHGE	0.931982
GLV	0.932038
RLV	0.887002
SZE	0.865687
LZE	0.995665
GLN	0.996621
ZSN	0.994426
ZP	0.901558
LGZE	0.934327
HGZE	0.966277
SZLGE	0.906793
SZHGE	0.971298
LZLGE	0.987982
LZHGE	0.980607
GLV	0.915213
ZSV	0.869444
Coarseness	0.989801
Contrast	0.889829
Busyness	0.996056
Complexity	0.862377
Strength	0.983378
energy of LLL decomposition	0.997769
h-entropy of LLL decomposition	0.919817
kurtosis of LLL decomposition	0.988672
max of LLL decomposition	0.902784
MAD of LLL decomposition	0.997536
mean of LLL decomposition	0.998173
media of LLL decomposition	0.991304
range of LLL decomposition	0.910254
skewness of LLL decomposition	0.998152
RMS of LLL decomposition	0.992002

SD of LLL decomposition	0.998525
h-uniformity of LLL decomposition	0.842496
var of LLL decomposition	0.998282
h-mean of LLL decomposition	0.997494
h-var of LLL decomposition	0.998259
h-skewness of LLL decomposition	0.9919
h-kurtosis of LLL decomposition	0.988398
Energy of LLL decomposition	0.802191
Contrast of LLL decomposition	0.94518
Entropy of LLL decomposition	0.95217
Homogeneity of LLL decomposition	0.955429
Correlation of LLL decomposition	0.977369
SumAverage of LLL decomposition	0.912623
Variance of LLL decomposition	0.968695
Dissimilarity of LLL decomposition	0.957703
SRE of LLL decomposition	0.898964
LRE of LLL decomposition	0.888046
GLN of LLL decomposition	0.996686
RLN of LLL decomposition	0.994987
RP of LLL decomposition	0.890884
HGRE of LLL decomposition	0.908266
SRHGE of LLL decomposition	0.896871
LRHGE of LLL decomposition	0.965136
GLV of LLL decomposition	0.852296
RLV of LLL decomposition	0.80441
SZE of LLL decomposition	0.856874
LZE of LLL decomposition	0.980083
GLN of LLL decomposition	0.996447
ZSN of LLL decomposition	0.993716
ZP of LLL decomposition	0.934449
HGZE of LLL decomposition	0.885937
SZHGE of LLL decomposition	0.904308
LZLGE of LLL decomposition	0.805103
LZHGE of LLL decomposition	0.982929
GLV of LLL decomposition	0.857606
ZSV of LLL decomposition	0.966036
Coarseness of LLL decomposition	0.919693
Contrast of LLL decomposition	0.947791
Busyness of LLL decomposition	0.95528
Complexity of LLL decomposition	0.908503
Strength of LLL decomposition	0.992419
energy of HLL decomposition	0.966317
mean of HLL decomposition	0.936943
skewness of HLL decomposition	0.911355

SD of HLL decomposition	0.911377
var of HLL decomposition	0.874897
gauss-fitting-a of HLL decomposition	0.97965
h-var of HLL decomposition	0.874415
h-skewness of HLL decomposition	0.882314
Energy of HLL decomposition	0.902656
Contrast of HLL decomposition	0.90271
Entropy of HLL decomposition	0.951418
Homogeneity of HLL decomposition	0.948129
Variance of HLL decomposition	0.887974
Dissimilarity of HLL decomposition	0.945621
SRE of HLL decomposition	0.917959
LRE of HLL decomposition	0.845774
GLN of HLL decomposition	0.999062
RLN of HLL decomposition	0.996559
RP of HLL decomposition	0.88643
LRHGE of HLL decomposition	0.861416
LZE of HLL decomposition	0.993756
GLN of HLL decomposition	0.99694
ZSN of HLL decomposition	0.996417
ZP of HLL decomposition	0.934602
LZLGE of HLL decomposition	0.9926
LZHGE of HLL decomposition	0.994144
GLV of HLL decomposition	0.947411
ZSV of HLL decomposition	0.971422
Coarseness of HLL decomposition	0.985897
Contrast of HLL decomposition	0.916687
Busyness of HLL decomposition	0.997498
Complexity of HLL decomposition	0.95259
Strength of HLL decomposition	0.991233
energy of LHL decomposition	0.974602
mean of LHL decomposition	0.948035
skewness of LHL decomposition	0.911701
SD of LHL decomposition	0.91171
var of LHL decomposition	0.87782
gauss-fitting-a of LHL decomposition	0.97899
gauss-ficting-c of LHL decomposition	0.826675
h-var of LHL decomposition	0.87762
Energy of LHL decomposition	0.860909
Contrast of LHL decomposition	0.888313
Entropy of LHL decomposition	0.929676
Homogeneity of LHL decomposition	0.928625
Variance of LHL decomposition	0.868579
Dissimilarity of LHL decomposition	0.930356

SRE of LHL decomposition	0.875174
GLN of LHL decomposition	0.99886
RLN of LHL decomposition	0.99729
RP of LHL decomposition	0.827809
LRHGE of LHL decomposition	0.88662
LZE of LHL decomposition	0.993359
GLN of LHL decomposition	0.997357
ZSN of LHL decomposition	0.996757
ZP of LHL decomposition	0.918711
LZLGE of LHL decomposition	0.992311
LZHGE of LHL decomposition	0.993604
GLV of LHL decomposition	0.941475
ZSV of LHL decomposition	0.961622
Coarseness of LHL decomposition	0.987206
Contrast of LHL decomposition	0.887686
Busyness of LHL decomposition	0.997286
Complexity of LHL decomposition	0.937187
Strength of LHL decomposition	0.990126
energy of HHL decomposition	0.964747
mean of HHL decomposition	0.930786
skewness of HHL decomposition	0.916853
SD of HHL decomposition	0.916841
var of HHL decomposition	0.884577
gauss-fitting-a of HHL decomposition	0.986368
h-var of HHL decomposition	0.8841
Energy of HHL decomposition	0.926452
Contrast of HHL decomposition	0.903872
Entropy of HHL decomposition	0.953518
Homogeneity of HHL decomposition	0.946636
Variance of HHL decomposition	0.907394
Dissimilarity of HHL decomposition	0.938717
SRE of HHL decomposition	0.941471
LRE of HHL decomposition	0.871068
GLN of HHL decomposition	0.998521
RLN of HHL decomposition	0.996739
RP of HHL decomposition	0.914242
LRHGE of HHL decomposition	0.882018
SZE of HHL decomposition	0.876771
LZE of HHL decomposition	0.995333
GLN of HHL decomposition	0.996698
ZSN of HHL decomposition	0.996926
ZP of HHL decomposition	0.880294
HGZE of HHL decomposition	0.871541
LZLGE of HHL decomposition	0.994695

LZHGE of HHL decomposition	0.99509
GLV of HHL decomposition	0.963691
ZSV of HHL decomposition	0.963332
Coarseness of HHL decomposition	0.993251
Contrast of HHL decomposition	0.856953
Busyness of HHL decomposition	0.998106
Complexity of HHL decomposition	0.911276
Strength of HHL decomposition	0.993997
energy of LLH decomposition	0.995549
h-entropy of LLH decomposition	0.879108
kurtosis of LLH decomposition	0.883795
max of LLH decomposition	0.82729
MAD of LLH decomposition	0.987233
mean of LLH decomposition	0.984923
media of LLH decomposition	0.951924
min of LLH decomposition	0.842137
range of LLH decomposition	0.894423
skewness of LLH decomposition	0.98085
RMS of LLH decomposition	0.908599
SD of LLH decomposition	0.980785
h-uniformity of LLH decomposition	0.893133
var of LLH decomposition	0.976641
gauss-fitting-a of LLH decomposition	0.988735
gauss-ficting-c of LLH decomposition	0.864792
h-mean of LLH decomposition	0.973736
h-var of LLH decomposition	0.976567
h-skewness of LLH decomposition	0.910808
h-kurtosis of LLH decomposition	0.885106
Energy of LLH decomposition	0.948378
Contrast of LLH decomposition	0.910648
Entropy of LLH decomposition	0.979235
Homogeneity of LLH decomposition	0.97835
Correlation of LLH decomposition	0.931595
Variance of LLH decomposition	0.910429
Dissimilarity of LLH decomposition	0.958704
SRE of LLH decomposition	0.964479
LRE of LLH decomposition	0.925473
GLN of LLH decomposition	0.999035
RLN of LLH decomposition	0.997715
RP of LLH decomposition	0.948323
SRHGE of LLH decomposition	0.87073
LRHGE of LLH decomposition	0.915558
GLV of LLH decomposition	0.886719
LZE of LLH decomposition	0.997087

GLN of LLH decomposition	0.997221
ZSN of LLH decomposition	0.997174
ZP of LLH decomposition	0.967654
HGZE of LLH decomposition	0.821329
LZLGE of LLH decomposition	0.997102
LZHGE of LLH decomposition	0.996989
GLV of LLH decomposition	0.974244
ZSV of LLH decomposition	0.984125
Coarseness of LLH decomposition	0.991726
Contrast of LLH decomposition	0.926833
Busyness of LLH decomposition	0.997859
Complexity of LLH decomposition	0.959033
Strength of LLH decomposition	0.990146
energy of HLH decomposition	0.953666
mean of HLH decomposition	0.908453
skewness of HLH decomposition	0.896224
RMS of HLH decomposition	0.84826
SD of HLH decomposition	0.896208
var of HLH decomposition	0.858801
gauss-fitting-a of HLH decomposition	0.982025
h-var of HLH decomposition	0.858764
h-skewness of HLH decomposition	0.846675
Energy of HLH decomposition	0.907008
Contrast of HLH decomposition	0.858177
Entropy of HLH decomposition	0.931362
Homogeneity of HLH decomposition	0.935488
Variance of HLH decomposition	0.858249
Dissimilarity of HLH decomposition	0.91498
SRE of HLH decomposition	0.917305
LRE of HLH decomposition	0.857979
GLN of HLH decomposition	0.998728
RLN of HLH decomposition	0.996689
RP of HLH decomposition	0.894327
LRHGE of HLH decomposition	0.85921
LZE of HLH decomposition	0.990532
GLN of HLH decomposition	0.995882
ZSN of HLH decomposition	0.995604
ZP of HLH decomposition	0.882497
LZLGE of HLH decomposition	0.989938
LZHGE of HLH decomposition	0.990057
GLV of HLH decomposition	0.961524
ZSV of HLH decomposition	0.969887
Coarseness of HLH decomposition	0.99285
Contrast of HLH decomposition	0.856914

Busyness of HLH decomposition	0.998085
Complexity of HLH decomposition	0.923876
Strength of HLH decomposition	0.989995
energy of LHH decomposition	0.966975
mean of LHH decomposition	0.926855
skewness of LHH decomposition	0.90719
SD of LHH decomposition	0.907225
var of LHH decomposition	0.879946
gauss-fitting-a of LHH decomposition	0.984814
h-var of LHH decomposition	0.880204
Energy of LHH decomposition	0.864606
Contrast of LHH decomposition	0.846749
Entropy of LHH decomposition	0.908112
Homogeneity of LHH decomposition	0.908598
Variance of LHH decomposition	0.848344
Dissimilarity of LHH decomposition	0.895073
SRE of LHH decomposition	0.873556
GLN of LHH decomposition	0.99902
RLN of LHH decomposition	0.997384
RP of LHH decomposition	0.817876
LZE of LHH decomposition	0.993058
GLN of LHH decomposition	0.995917
ZSN of LHH decomposition	0.996159
ZP of LHH decomposition	0.860337
LZLGE of LHH decomposition	0.992873
LZHGE of LHH decomposition	0.992163
GLV of LHH decomposition	0.955379
ZSV of LHH decomposition	0.957566
Coarseness of LHH decomposition	0.993945
Contrast of LHH decomposition	0.816726
Busyness of LHH decomposition	0.997347
Complexity of LHH decomposition	0.889471
Strength of LHH decomposition	0.99008
energy of HHH decomposition	0.968515
h-entropy of HHH decomposition	0.801643
mean of HHH decomposition	0.934869
skewness of HHH decomposition	0.926746
SD of HHH decomposition	0.926716
var of HHH decomposition	0.902951
gauss-fitting-a of HHH decomposition	0.98671
h-mean of HHH decomposition	0.892803
h-var of HHH decomposition	0.902907
Energy of HHH decomposition	0.884366
Contrast of HHH decomposition	0.918476

Entropy of HHH decomposition	0.954516
Homogeneity of HHH decomposition	0.934396
Variance of HHH decomposition	0.919126
Dissimilarity of HHH decomposition	0.94892
SRE of HHH decomposition	0.932343
LRE of HHH decomposition	0.823468
GLN of HHH decomposition	0.998401
RLN of HHH decomposition	0.996792
RP of HHH decomposition	0.880042
SRHGE of HHH decomposition	0.800772
LZE of HHH decomposition	0.996404
GLN of HHH decomposition	0.997463
ZSN of HHH decomposition	0.997185
ZP of HHH decomposition	0.937842
LZLGE of HHH decomposition	0.995784
LZHGE of HHH decomposition	0.996034
GLV of HHH decomposition	0.944919
ZSV of HHH decomposition	0.968715
Coarseness of HHH decomposition	0.9907
Contrast of HHH decomposition	0.883982
Busyness of HHH decomposition	0.998715
Complexity of HHH decomposition	0.909444
Strength of HHH decomposition	0.993045
